# Comparison of the Effects of Anaesthesia Methods Used in Caesarean Delivery on Neonatal Cerebral and Renal Oxygenation: A Randomised Controlled Trial

**DOI:** 10.3390/jcm13030873

**Published:** 2024-02-02

**Authors:** Ulku Arslan, Nilgun Kavrut Ozturk, Ali Sait Kavakli, Hatice Ozge Dagdelen

**Affiliations:** 1 Department of Anesthesiology and Reanimation, University of Health Sciences, Antalya Training and Research Hospital, 07100 Antalya, Turkey; kavrut@yahoo.com (N.K.O.); oszgedagdelen@gmail.com (H.O.D.); 2 Department of Anesthesiology and Reanimation, Istinye University Faculty of Medicine, 34010 Istanbul, Turkey; alisaitkavakli@hotmail.com

**Keywords:** anaesthesia, near-infrared spectroscopy, regional oxygen saturation, newborn

## Abstract

**Background**: During a newborn’s adaptation to extrauterine life, many changes take place that are influenced by various factors. The type of delivery and anaesthesia strategy utilised during labour can modify these adaptive modifications. In this regard, this study was designed to compare the effects of general and spinal anaesthesia on cerebral and renal oxygenation after elective caesarean deliveries. **Methods**: This randomised controlled study comprised sixty parturient women who were over 18 years old and had a gestational age between 37 and 41 weeks. All participants had an ASA (American Society of Anesthesiologists) classification of II. Neonatal cerebral (CrSO_2_) and renal (RrSO_2_) regional oxygen saturations were assessed using near-infrared spectroscopy. Additionally, the 1st–5th min Apgar scores, preductal and postductal peripheral oxygen saturation (SpO_2_), and perfusion index were recorded in both the general anaesthesia and spinal anaesthesia groups. **Results**: There was no statistically significant difference between the two groups in terms of CrSO_2_ or RrSO_2_ values. The values of CrSO_2_ and RrSO_2_ in both groups showed a significant rise from the 10th to the 15th min, respectively. **Conclusions**: General and spinal anaesthesia techniques used for cesarean delivery have similar effects on neonatal cerebral and renal oxygenation.

## 1. Introduction

Complex and important physiological changes that are a part of the neonatal adaptation process to extrauterine life can depend on maternal and environmental factors. The type of delivery, as well as the use of labour anaesthesia, may be factors that contribute to poor neonatal adaptation [1]. The selection of the anaesthesia technique, whether it be general anaesthesia or spinal anaesthesia, is determined by various criteria, including the urgency of the surgery, the indication for operational delivery, the clinical status of the patient, and the attribution of the obstetrician. The anaesthesia used during a caesarean section should emphasise the safety and comfort of the mother, the optimal health outcome of the newborn, and the best working conditions for the surgeon [2].

Measuring and monitoring peripheral oxygen saturation (SpO_2_) and regional tissue oxygen saturation (rSO_2_) can be beneficial regarding the early detection of hypoxia and hyperoxia, which can impact the process of postnatal adaptation [3]. Near-infrared spectroscopy (NIRS) allows continuous, non-invasive monitoring of tissue oxygenation and perfusion [4]. Although there have been several studies demonstrating early changes in neonates’ tissue oxygen saturation after birth, data regarding follow-up monitoring of cerebral (CrSO_2_) and renal (RrSO_2_) tissue oxygenation involving the whole adaptation process is inadequate [5,6]. Furthermore, the relationship between the anaesthesia techniques used during a caesarean section and the postpartum oxygenation of a newborn remains uncertain. Therefore, the aim of this study was to investigate the impact of spinal and general anaesthesia on the cerebral regional oxygen saturation (CrSO_2_) and renal regional oxygen saturation (RrSO_2_) of newborns during the early neonatal period.

## 2. Methods

The randomized controlled study was conducted in compliance with the Declaration of Helsinki, which was approved by the Institutional Ethics Committee of the Antalya Training and Research Hospital (approval number 1/1, dated 5 January 2017), and registered at Clinicaltrials.gov (no. NCT03389139). The main objective of this study was to investigate the differences in neonatal cerebral regional oxygen saturation (CrSO_2_). The secondary aim of this study was to measure variations in RrSO_2_, Apgar scores, preductal and postductal SpO_2_, perfusion index, and body temperature of the babies. Prior to their enrollment in the study, all participants provided written informed consent. The risks and benefits of both anaesthesia methods (spinal or general anaesthesia) were explained to the patients. Participants who were at least 18 years old, classified as American Society of Anaesthesiologists (ASA) I–II, and scheduled for elective caesarean delivery were randomly assigned to one of two groups using a method that involved drawing numbers placed in envelopes: Group SA (those who received spinal anaesthesia) and Group GA (those who received general anaesthesia). Patients who decided to choose an alternative anaesthesia technique after randomization were excluded from the study. Candidates with diabetes, hypertension, renal failure, hepatic failure, congestive heart failure, heart valve disease, asthma, or chronic obstructive pulmonary disease were excluded from the study. Furthermore, individuals who were experiencing preeclampsia, eclampsia, preterm, postterm, or multiple pregnancies, as well as those with an expected neonatal weight below 2500 g or above 4000 g, Rh incompatibility, or foetal or placental abnormalities were also excluded. Neonates who required postnatal resuscitation and/or oxygen support after randomization were not included in the study.

All caesarean sections were performed by two senior obstetrician gynecologists. The initial hemodynamic monitoring, including non-invasive blood pressure, three-lead electrocardiography, and pulse oximetry, was assessed soon after admission to the operating room, and the recorded values were noted as the baseline values. Group SA underwent spinal anaesthesia while in a seated position, and we utilised 0.5% hyperbaric bupivacaine at the L4–5 level. The level of sensory block was assessed with a pinprick test, and the intensity of the motor block was tested using the Bromage scale. A sensory block level of T4–T6 was considered adequate for surgical anaesthesia. A sensory block level of T10 or lower was considered a failure, and such patients were excluded from the study. A nasal cannula was used to deliver perioperative oxygen support in order to maintain a target SpO_2_ level of 94–98% as necessary. The hemodynamic parameters of the parturient were monitored at 3, 5, and 10 min following spinal anaesthesia.

In Group GA, anaesthesia induction was achieved with 2 mg/kg propofol and 0.6 mg/kg rocuronium following preoxygenation. Controlled ventilation was provided with a tidal volume of 6–8 mL/kg of ideal weight and a positive end-expiratory pressure of 6–8 cm H_2_O. The expiratory ratio was adjusted to ensure an end tidal carbon dioxide value of 30–35 mmHg. The anaesthesia was maintained using a mixture of 50% oxygen and 50% air, with the aim of maintaining the SpO_2_ level between 94–98%, and 1 MAC (minimum of alveolar concentration) of sevoflurane was administered. An amount of 0.15 mg/kg rocuronium was added when necessary. After the delivery and clamping the umbilical cord, 1–2 mg/kg intravenous fentanyl was administered to the parturient. Following the surgery, the residual neuromuscular block was reversed using neostigmine 0.05 mg/kg and atropine 0.02 mg/kg.

**Figure 1:**
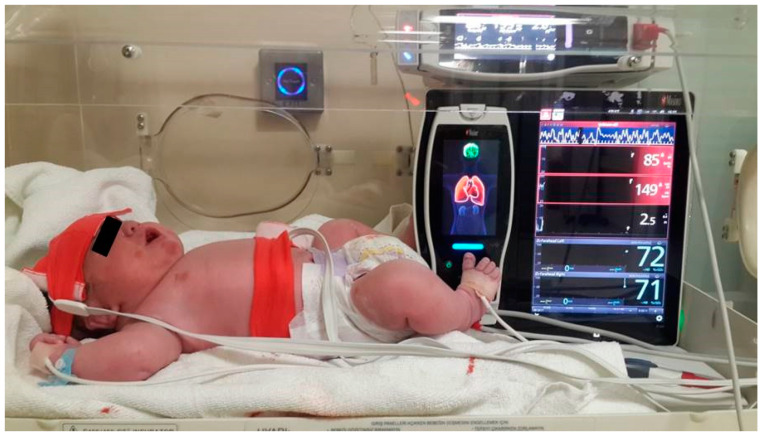
Monitoring of the newborn. On the large screen at the bottom, postductal SpO_2_, heart rate, perfusion index, cerebral and renal regional oxygen saturation can be observed; on the small screen at the top, preductal SpO_2_ and heart rate are displayed.

A staff nurse immediately transported all neonates to the baby care station, and used heated surgical drapes to dry them. Neonatal NIRS probes (O3TM, Masimo, Irvine, CA, USA) were placed at the frontal region of the head, as well as on the right kidney region at the T12–L2 level, by a blinded researcher. Measurements of CrSO_2_ and RrSO_2_ were taken at 3 min (baseline) and 5 min after birth, and then at 5-min intervals for the following 60 min. A pulse oximetry probe (M-LNCS NeoPt-500, MasimoSET, Masimo, Irvine, CA, USA) attached to the right hand was used to measure preductal SpO_2_, HR, and perfusion. Postductal SpO_2_ measurements were evaluated with a probe attached to the right foot (Figure 1). Body temperature measurements were obtained from the right axillary region (Omron Eco Temp Smart, MC 341-E, OMRON Healthcare Europe B.V.) The neonates birth weight, 1 and 5-min Apgar scores, HR, body temperature, preductal and postductal SpO_2_, and perfusion index values measured at postnatal 3 min, 5 min, and at 5-min intervals for 60 min were recorded.

### Statistical Analysis

Sample size for the study was calculated using G-power (G*Power 3.1, Düsseldorf, Germany) version 3.1.9.2 software. A pilot study of 15 patients per group suggested a 10% relative change of CrSO_2_ in the spinal anaesthesia group as compared to the general anaesthesia group was significant. Consequently, a minimum necessary sample size was estimated at 27 patients in each group through using a two-sided test with 95% power and 5% significance level. Therefore, 35 patients per group were included to compensate for dropouts.

Statistical analysis was performed using SPSS version 24 statistical software (SPSS Inc., Chicago, IL, USA). Continuous variables were presented as mean ± standard deviation (SD) and 95%CI, and ordinal variables as median and interquartile range (IQR) values. Categorical variables were presented as the number (n) and percentage (%). Differences between the mean values for normally distributed variables were compared using the Student’s *t*-test. Non-normally distributed variables were compared with the Mann-Whitney U-test and Wilcoxon rank sum test. The Chi-square test and Fisher Exact test were used for categorical data where appropriate. Repeated measures of variance (ANOVA) were used to test for any change in baseline values and data obtained at each time point. Pearson and Spearman correlation coefficients were used for correlation analyses. A value of *p* < 0.05 was considered statistically significant.

## 3. Results

**Figure 2:**
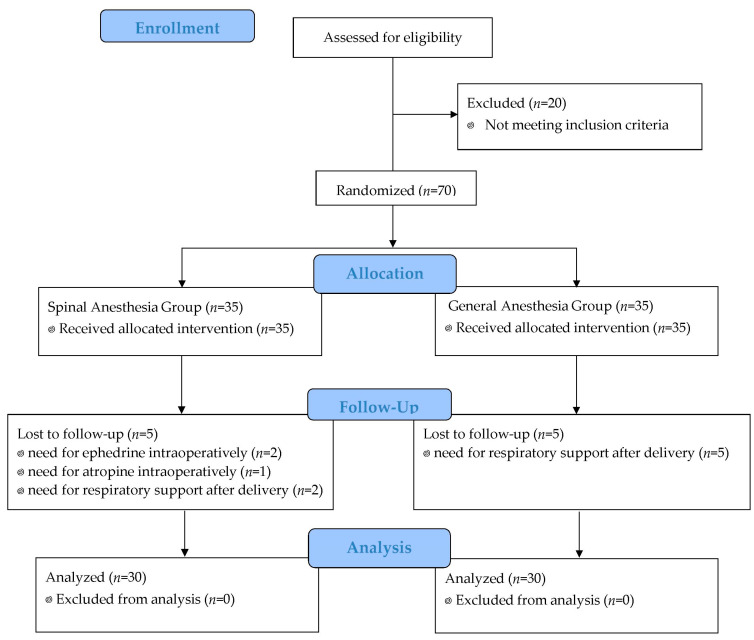
Consolidated Standards of Reporting Trials (CONSORT) flow diagram.

**Figure 3:**
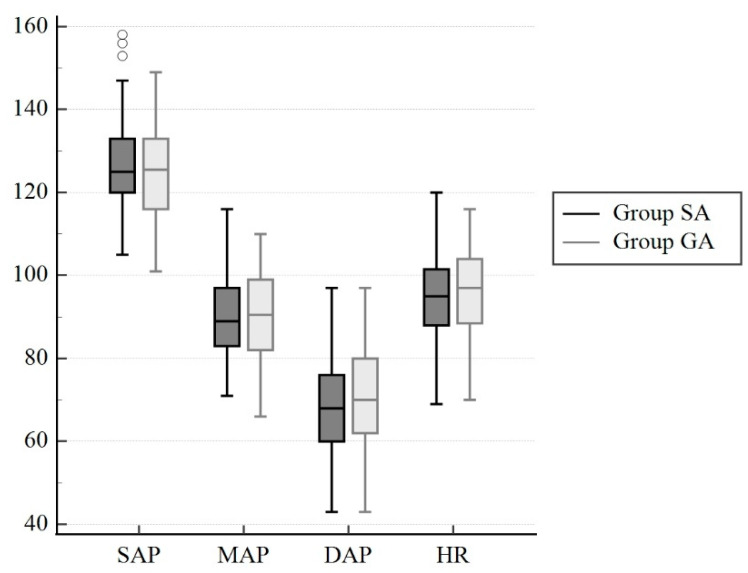
Box plots of intraoperative hemodynamic variables for parturients who received spinal or general anesthesia. SAP, systolic arterial pressure; DAP, diastolic arterial pressure, MAP, mean arterial pressure; HR, heart rate.

A total of 90 parturients were evaluated for eligibility in the study, and 20 were excluded due to not meeting the inclusion criteria. Therefore, 70 patients were enrolled in the study. One participant in Group SA, as well as two participants in Group GA, withdrew from the study after randomization Three parturients in group SA were switched to general anaesthesia due to failed spinal anesthesia or a massive intraoperative hemorrhage (>2000 cc). One neonate in Group SA and three neonates in Group GA required respiratory support after delivery. Finally, data from the remaining 60 parturients and neonates were analysed for the study (Figure 2). All participants had an ASA (American Society of Anesthesiologists) classification of II. Demographic data for the parturients and neonates are presented in Table 1. In Group SA, the level of sensory block reached T4 in all cases. The mean propofol consumption in Group GA was 187 ± 11 mg, while the mean rocuronium bromide consumption was 44 ± 5 mg. Caesarean sections were performed without any complications in both study groups. Intraoperative blood loss was 473 ± 53 mL in Group SA and 486 ± 44 mL in Group GA (*p* = 0.979). There were no statistically significant differences observed in the systolic, diastolic, and mean arterial pressures, as well as heart rate, during the first 10 min following anaesthesia induction in parturiens who underwent either spinal or general anaesthesia (*p* = 0.546, *p* = 0.478, *p* = 0.679, *p* = 0.579, respectively) (Figure 3).

**Figure 4:**
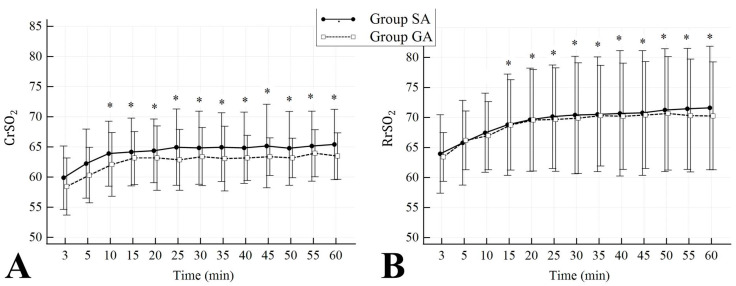
Changes over time in neonatal cerebral (**A**) and renal (**B**) regional oxygen saturation values in both groups. CrSO_2_, cerebral regional oxygen saturation; RrSO_2_, renal regional oxygen saturation. * *p* < 0.05 for cerebral and renal rSO_2_ values compared to 3-min (baseline) in both groups.

Mean neonatal CrSO_2_ values were 64.2 ± 3.2 in Group SA and 62.5 ± 2.6 in Group GA. The mean RrSO_2_ values were 69.4 ± 5 in Group SA and 68.9 ± 4.4 in Group GA. Both groups showed no significant differences in CrSO_2_ values at the 5th minute when compared to the 3rd minute (baseline). In contrast, it was observed that the CrSO_2_ levels at and after 10 min significantly increased compared to the baseline (Figure 4A). Although RrSO_2_ values were similar at 3, 5, and 10 min, they were significantly higher in both groups at and after 15 min when compared to the 3rd minute (baseline) (Figure 4B). Comparison of neonatal data is presented in Table 2. There were no significant differences in terms of preductal SpO_2_, postductal SpO_2_, HR, body temperature, and perfusion index in Group SA when compared to Group GA (*p* = 0.268, *p* = 0.068, *p* = 0.947, *p* = 0.759, *p* = 0.580, respectively).

In Group SA, positive correlations were observed between preductal SpO_2_ and CrSO_2_ (*p* = 0.001, r = 0.423), as well as between postductal SpO_2_ and RrSO_2_ (*p* = 0.001, r = 0.454). Likewise, there were also significant positive correlations between preductal SpO_2_ and CrSO_2_ (*p* = 0.001, r = 0.384), as well as postductal SpO_2_ and RrSO_2_ (*p* = 0.001, r = 0.408) in Group GA.

## 4. Discussion

The results of our study revealed that both spinal and general anaesthesia have significant impacts on the oxygenation of infant cerebral and renal tissues. Both the Neonatal CrSO_2_ and RrSO_2_ levels showed a similar increase over time with both anaesthesia procedures. The results of our study revealed that there were positive correlations between the CrSO_2_ and the preductal SpO_2_ values in newborns. Additionally, there were correlations between the RrSO_2_ values and the postductal SpO_2_ values. Furthermore, we found correlations between both RrSO_2_ and CrSO_2_ values and body temperature in both groups.

The results of the studies investigating the impact of vaginal delivery versus caesarean section on neonatal SpO_2_ values are inconclusive, as certain studies have indicated lower SpO_2_ levels in the first minutes following a caesarean section [7,8], while others revealed no difference [9,10]. Although there have been no previous studies that specifically examine the impact of anaesthesia methods used in caesarean sections on infant peripheral SpO_2_ values, Özgen et al. [11] conducted a comparison of different anaesthesia techniques on the rSO_2_ values of newborns, and detected that preductal SpO_2_ values were lower in the general anaesthesia group when compared to the regional anaesthesia. However, their measurements involved just the first 2.5 min after delivery, with no further information in the subsequent time period. In order to achieve reliable results, we obtained rSO_2_ measurements between 3 and 60 min in our study, and observed no significant differences between the general and spinal anaesthesia groups in terms of preductal or postductal SpO_2_ values. In the group receiving general anaesthesia, the management of hemodynamics and ventilation was more reliable. Nevertheless, the administration of hypnotic and narcotic medicines to the newborn before delivery may lead to respiratory depression and low SpO_2_ after birth, both in the short and long term. Evans et al. [12] proposed that the occurrence of foetal depression following elective caesarean section procedures conducted under general anaesthesia could be attributed to the transmission of anaesthetic drugs through the placenta, whereas the most likely cause of hypoxia in newborns after a caesarean section with regional anaesthesia is hypotension and respiratory failure, which may have developed due to sympathetic blockade and an increased block level in the mother [13]. In our study, we did not carry out a blood analysis to measure the concentrations of sedative analgesic drugs in newborns delivered under general anaesthesia; however, we found no significant difference in the Apgar evaluation or the preductal and postductal SpO_2_ values during the clinical observation. No complications leading to foetal depression due to block level and hemodynamics were observed in the spinal anaesthesia group. In addition, we administered oxygen support to maintain the SpO_2_ goal of 94–98% in both groups. The absence of any difference in the preductal or postductal SpO_2_ values of the newborns between the two groups in our study can be attributed to the similarity in SpO_2_ goals, as well as to the diligent follow-up of maternal oxygenation. In light of these data, it was thought that the protocol applied in our study in the general anaesthesia group did not cause depression in the newborn; therefore, similar results were obtained with spinal anaesthesia administered under optimal conditions in terms of tissue perfusion and Apgar evaluations of newborns.

This study found that postpartum CrSO_2_ and RrSO_2_ values increased during the first hour, regardless of the anaesthesia procedures used. Under both anaesthesia procedures, there was a considerable increase in CrSO_2_ values starting from 10 min, and in RrSO_2_ values starting from 15 min, when compared to the values at 3 min. Ilves et al. [14] stated that significant changes in cerebral and visceral blood flow in the first hours of life were related to the neonatal adaptation process after birth. In certain studies, it was observed that there was a notable increase in cerebral blood flow within the initial 12 h of labour [15], whereas the rise in renal arterial blood flow was comparatively slower [16]. Montaldo et al. [6] reported that the RrSO_2_ and mesenteric rSO_2_ values were observed to be lower than the CrSO_2_ values during the first 7 min of life. It was also observed that the rise in RrSO_2_ and mesenteric rSO_2_ values is slower compared to CrSO_2_ values. The authors emphasised that the differences may arise due to concomitant variations between preductal and postductal SpO_2_ values. Urlesberger et al. [17] found that the rSO_2_ values showed a faster increase within the first 7 min of life compared to the CrSO_2_ values. Additionally, the fractional tissue oxygen extraction reached a plateau earlier than peripheral tissues. Remaining consistent with these studies, our study also revealed that CrSO_2_ values reached a plateau earlier than RrSO_2_ values. Previous studies have also shown that in the first few minutes of the postpartum period, there was a progressive rise in CrSO_2_ values over a certain period. The common findings of these studies demonstrate a progressive increase in CrSO_2_ value during the first 7–10 min after birth [17,18,19,20,21,22,23]. However, no definite consensus exists on the course of CrSO_2_ after 10 min. In our study, CrSO_2_ values reached a plateau after rising progressively over the first 10 min in both groups. The similarity in mean CrSO_2_ values between the two groups suggests that this period was not affected by the anaesthesia method.

Physiologically, closure of the ductus arteriosus begins immediately following birth, and is completed within 24–72 h. In cases of patent ductus arteriosus (PDA), if the left-to-right shunt is greater than 50% of the left ventricular output, the effective systemic blood flow decreases despite a continued increase in left ventricular output [24]. Increased left ventricular output due to PDA is also accompanied by an increase in stroke volume. Therefore, if there is no left-to-right shunt in the ductus arteriosus in newborns, there may be a temporary decrease in the left ventricular output, which may result in reduced carotid blood flow. This mechanism has been proposed as a possible explanation for the lower CrSO_2_ values immediately after birth in newborns with no PDA shunt [25]. Vanderhaegen et al. [26] reported a rapid rise in CrSO_2_ values starting 5 min after surgical closure of PDA that lasts for 20 min. In our study, mean values of postpartum CrSO_2_ increased for the first 10 min and showed no significant increase thereafter. However, detection of PDA was beyond the scope of this study.

There may be a difference between preductal and postductal SpO_2_ values due to the patency of the ductus arteriosus within the first few minutes postpartum, which may explain the discrepancy between CrSO_2_ and RrSO_2_ values. In a study of preterm neonates with PDA exhibiting hemodynamic symptoms, Chock et al. [27] showed that PDA was associated with low RrSO_2_ values, while CrSO_2_ values were unaffected. The authors speculated that CrSO_2_ was protected by the physiologic capacity to autoregulate cerebral blood flow. In contrast, Bailey et al. [5] reported that RrSO_2_ values were higher than CrSO_2_ values during the first 24 h of life. Supporting this finding, Ilves et al. [14] reported that flow rates measured in the anterior cerebral, basilar, middle cerebral, and inferior cerebral arteries were lower than the renal arterial flow rate within the first few hours after birth. Postnatal cerebral blood flow and oxygen delivery are determined by cerebral vascular resistance, which is regulated by variables such as cardiac output variability associated with ductal closure time, increased blood flow due to vasodilation in major cerebral arteries, blood oxygen content, and increased cerebral metabolism. Ductal closure time, dynamic changes in cerebral vascular vessel diameter, and progressively increasing cerebral metabolism may lead to variations in CrSO_2_ value in terms of different clinical reports and other visceral organs [25].

In our study, the absence of a difference between preductal and postductal SpO_2_ values in both groups suggests physiological closure of the ductus arteriosus or a clinically insignificant shunt. Higher RrSO_2_ values compared to CrSO_2_ values in both groups may be due to the difference in flow rates as reported by Ilves et al. [14]. In addition, the similarity between the CrSO_2_ and rSO_2_ values obtained in both study groups may indicate that the anaesthesia techniques had no effect on neonatal renal and cerebral oxygenation. Further studies investigating RrSO_2_ and CrSO_2_ values in neonates without an echocardiographically confirmed PDA shunt are needed to reveal the effect of a physiologic shunt on organ perfusion.

Regional oxygen saturation levels acquired immediately after birth may be unreliable in 50% of newborns due to wet skin, restlessness, or crying [17]. Similarly, Watanabe et al. [28] reported that stable CrSO_2_ signals were obtained in only 59% of newborns, while stable SpO_2_ signals could not be measured in any of the newborns at measurements obtained 1 min after birth. Stable CrSO_2_ and SpO_2_ measurements could be achieved after 3 min at rates of 97% and 78%, respectively. These measurement artefacts may lead to misinterpretations in the evaluating systemic and tissue oxygen saturations. The compatibility between the monitored regional oxygen saturation curves and the pulse rate, as well as the proper amplitude, can provide insight into the accuracy of the obtained numerical saturation values [29]. During our study, we paid attention to taking all measurement records while the optimum saturation curve was present.

Our study has several limitations. Firstly, the evaluation of patent ductus arteriosus in the neonates was not assessed using echocardiography. Although our findings did not indicate the presence of a clinically important shunt in the ductus arteriosus, we believe that the absence of flow, or the presence of a flow, through the ductus arteriosus that can be clinically negligible should be confirmed by echocardiography. Secondly, some rSO_2_ measurements may be inaccurate due to signal pollution, especially when measuring RrSO_2_. Thompson et al. have shown that meconium especially may have a light absorbance spectrum that can cause errors in the measurement of splanchnic rSO_2_ values [30]. Although meconium was not observed on any of the neonates in our study, it is not certain whether the vernix caseosa layer found on neonates causes light absorption. Thirdly, Spinal anesthesia was chosen as the method of regional anesthesia in our study. Cesarean sections can also be performed under epidural anesthesia and may have an effect on neonatal oxygenation [31,32]. Fourthly, dynamic parameters were not taken into account regarding hemodynamic monitoring during the intraoperative follow-up of the participants in our study. Monitoring dynamic parameters with continuous non-invasive hemodynamic monitoring may be important in terms of detecting dynamic parameters, such as detecting short-term hypotension attacks that may affect the well-being of the newborn [33]. Further studies are required to clarify this issue. Finally, the cerebral and renal adaptation of newborns may be influenced by blood pH, PaCO_2_, and hematocrit values, which were not investigated in our study, and the flow rates in the cerebral and renal arteries were not measured. Studies evaluating these parameters may confirm our data.

In conclusion, this study demonstrated that both general and spinal anaesthesia techniques used during caesarean sections have similar impacts on the cerebral and renal rSO_2_ levels of newborns. Our findings also revealed that RrSO_2_ values were higher than CrSO_2_ values during the first 60 min of life, and the increase in RrSO_2_ levels was lower compared to CrSO_2_. Further studies with a larger sample size are required to confirm the results of this study.

## Figures and Tables

**Table 1:** Demographic data of the parturients and neonates (Data are expressed as the mean ± standard deviation, median with interquartile range).

	Group SA (*n* = 30)	Group GA (*n* = 30)	Difference (95% CI)
Maternal age, years	30 ± 5	28 ± 5	−2.600 (−5.344 to 0.144)
Gestational age, weeks	38.8 ± 0.5	38.6 ± 0.4	−0.267 (−0.544 to 0.010)
Maternal BMI, kg/m^2^	27.2 ± 3.4	27.5 ± 3.2	0.370 (−1.388 to 2.128)
Maternal ASA status			
ASA II	30 (100)	30 (100)	0 (0 to 0)
Gender of neonates			
Male, *n* (%)	15 (50)	16 (51.6)	0.933 (0.552 to 1.575)
Female, *n* (%)	15 (50)	14 (48.3)	
Birth weight, g	3351 ± 290	3386 ± 244	35.333 (−103.215 to 173.881)
APGAR scores			
1 min	9 (9–10)	9 (9–9)	0 (−1 to 0)
5 min	10 (10–10)	10 (10–10)	0 (0 to 0)
Delivery time, min	5.8 ± 0.9	3.7 ± 0.5	−2.133 (−2.526 to −1.741)
Maternal preoperative hemoglobin, g/dL	11.9 ± 1.3	11.9 ± 1.1	0.060 (−0.550 to 0.670)

BMI: Body Mass Index, ASA: American Sociery of Anaeshesiologists.

**Table 2:** Comparison of the neonatal data between the study groups (Data are expressed as the mean ± standard deviation).

	Group SA (*n* = 30)	Group GA (*n* = 30)	Difference (95% CI)
Preductal SpO_2_ (%)	94.1 ± 3.9	93.7 ± 4.6	−0.341 (−0.945 to 0.262)
Postductal SpO_2_ (%)	92 ± 4.2	91.4 ± 5.3	−0.630 (−1.307 to 0.045)
Heart rate (beat/min)	152 ± 8	151 ± 9	−0.012 (−0.393 to 0.367)
Body temperature (°C)	36.5 ± 0.5	36.4 ± 0.6	−0.047 (−0.132 to 0.037)
Perfusion index (%)	2.2 ± 0.4	2.3 ± 0.6	0.070 (−0.174 to 0.314)

SpO_2_: Peripheral oxygen saturation, SA: Spinal anesthesia, GA: General anesthesia.

## Data Availability

All data generated or analyzed during this study are included in this article. Further inquiries can be directed to the corresponding author.
